# Mitonuclear Genetic Interactions in the Basidiomycete *Heterobasidion parviporum* Involve a Non-conserved Mitochondrial Open Reading Frame

**DOI:** 10.3389/ffunb.2021.779337

**Published:** 2021-12-14

**Authors:** Pierre-Henri Clergeot, Åke Olson

**Affiliations:** Department of Forest Mycology and Plant Pathology, Swedish University of Agricultural Sciences, Uppsala, Sweden

**Keywords:** heterokaryon, mitochondria, nucleus, mycelium, growth, cytonuclear

## Abstract

The mitochondrial and nuclear genomes of Eukaryotes are inherited separately and consequently follow distinct evolutionary paths. Nevertheless, the encoding of many mitochondrial proteins by the nuclear genome shows the high level of integration they have reached, which makes mitonuclear genetic interactions all the more conceivable. For each species, natural selection has fostered the evolution of coadapted alleles in both genomes, but a population-wise divergence of such alleles could lead to important phenotypic variation, and, ultimately, to speciation. In this study in the Basidiomycete *Heterobasidion parviporum*, we have investigated the genetic basis of phenotypic variation among laboratory-designed heterokaryons carrying the same pair of haploid nuclei, but a different mitochondrial genome. Radial growth rate data of thirteen unrelated homokaryotic parents and of their heterokaryotic offspring were combined with SNP data extracted from parental genome sequences to identify nuclear and mitochondrial loci involved in mitonuclear interactions. Two nuclear loci encoding mitochondrial proteins appeared as best candidates to engage in a genetic interaction affecting radial growth rate with a non-conserved mitochondrial open reading frame of unknown function and not reported apart from the Russulales order of Basidiomycete fungi. We believe our approach could be useful to investigate several important traits of fungal biology where mitonuclear interactions play a role, including virulence of fungal pathogens.

## Introduction

Mitochondria are functionally and genetically dynamic organelles essential to the eukaryotic cell (Burger et al., 2003; Henze and Martin, 2003; McBride et al., 2006). They are the center of aerobic production of energy, but also play a role in signaling between cellular components, cellular growth and differentiation, apoptosis, control of the cell cycle, disease, and aging (McBride et al., 2006; Bernhardt et al., 2014; Urbani and Babu, 2019). Although the majority of mitochondrial proteins are encoded by nuclear genes, mitochondria have their own self-replicating genome bearing phylogenetic evidence of prokaryotic ancestry (Gray et al., 2001). According to the endosymbiotic theory, mitochondria originate from the endocytosis of an alphaproteobacterium that took place about two billions years ago as an adaptation to an atmosphere richer in oxygen (Gray et al., 1999). Mitochondrial and nuclear genomes have subsequently coevolved throughout the evolutionary history of Eukaryota, following two major trends: one of reduction of the number of mitochondrial genes non-essential to the eukaryotic cell, the other of transfer to the nucleus of unique essential genes whose products can be imported from the cytosol to the mitochondrion without fitness cost (Kurland and Andersson, 2000). Mitochondrial genes encoding proteins involved in cellular respiration are highly conserved while other loci are more variable depending on the species, and some contribute to mitochondrial genome evolution and plasticity, like highly polymorphic, repetitive elements found within introns and intergenic regions (Burger et al., 2003). Consequently, conformation of the mitochondrial genome, its size, its degree of relatedness to its bacterial ancestor, its genes number, their order and their expression vary by species (Gray et al., 1999; Medina et al., 2020 for a recent review in fungi). Maintaining mitochondrial activity with a reduced genome implies multiple mitonuclear genetic interactions (Stojković and Dordević, 2017), but also requires nuclear and mitochondrial DNA permanently to coadapt despite their frequently different mutation rates (Allio et al., 2017; Sandor et al., 2018; Medina et al., 2020). Population-wise divergence of mitochondrial and nuclear coadapted alleles is predicted to lead to speciation due to Bateson-Dobzhansky-Muller incompatibility (Nguyen et al., 2020 and references therein).

The goal of this article is to show that harnessing natural intraspecific variation of coadapted mitochondrial and nuclear alleles in order to study genetics of mitonuclear interactions is possible. Natural variation in the mitochondrial genome of two species of the tree pathogen *Heterobasidion* is associated with the emergence of interspecific heterokaryotic hybrids with virulence that correlates with mitochondrial inheritance (Olson and Stenlid, 2001; Giordano et al., 2018). This observation suggests that, in self-incompatible Basidiomycetes species such as *Heterobasidion spp*., new combinations of mitochondrial and nuclear DNA formed at the heterokaryotic phase could drastically alter phenotype. Isolates of *Heterobasidion spp*. spend most of their life cycle as heterokaryotic mycelium (n + n) in host wood (Johannesson and Stenlid, 2004; Garbelotto and Gonthier, 2013). The homokaryotic (haploid) phase is short and exposed mainly to purifying and neutral selections: Basidiospores dispersal by the wind, their landing on an exposed area of a potential host, and the encounter of homokaryotic mycelia originating from different basidiocarps on host wood are largely stochastic. Mating is under the control of a unifactorial multiallelic genetic system (Holt et al., 1983), which, preceded by basidiospore dispersal, is supposed to favor outbreeding (Nieuwenhuis et al., 2013). It is therefore when mitochondrial and nuclear DNA of different homokaryotic isolates meet at the heterokaryotic phase that new mitonuclear combinations might emerge. If so, some degree of mitochondrial DNA-dependent phenotypic variation is expected at each generation of intraspecific heterokaryons as well.

Our recent work in *Heterobasdion parviporum* demonstrated and quantified mitochondrial genetic effects on two fitness proxies in heterokaryons and their parents (Clergeot et al., 2019). Our experimental design was based on heterokaryon synthesis (Olson and Stenlid, 2001), during which mitochondrial DNA is unparentally inherited while nuclei undergo bidirectional exchange, resulting in a pair of heterokaryons with the same haploid nuclei but a different mitochondrial genome (Xu and Wang, 2015). Heterokaryon synthesis was implemented with thirty homokaryotic parental isolates sampled at various locations in Eurasia and showing no population structure. We observed that variance of growth rate does not correlate with genetic distance in heterokaryons and that mitochondrial genetic effects account for 35% of phenotypic variance among homokaryons and 3% among heterokaryons (Clergeot et al., 2019). Heterokaryons are more fit than their parents in average, but not more than the fittest parents, suggesting heterozygosity and the masking of deleterious alleles at some of the nuclear loci involved in mitonuclear interactions in the offspring, rather than an intrinsic difference of fitness between the heterokaryons and their parents.

The present study is an investigation of the genetic basis of mitonuclear interactions in *Heterobasidion parviporum*: As phenotyping the heterokaryotic offspring of pairwise crossings of homokaryotic parents in controlled conditions brings a more reliable assessment of the variation resulting from combining different nuclear and mitochondrial alleles (Nguyen et al., 2020), we have revisited our data of heterokaryon variance for growth rate and parental genome sequence polymorphism (Clergeot et al., 2019) in order to identify candidate loci acting in *trans* across the two genomes. We have been able to single out this way two possible candidates for a nuclear locus encoding proteins involved in molecular trafficking to the mitochondrion (a mitochondrial carrier protein or a mitochondrial calcium uniporter), and a non-conserved mitochondrial locus of unknown function and whose distribution is limited to the Russulales order of Agaricomycetes. We believe that our results on mitonuclear genetics of growth rate in *H. parviporum* could pave the way to similar investigations of mitochondrial DNA-dependent variation of important traits e.g., virulence in fungal pathogens.

## Materials and Methods

### Fungal Isolates

Data from thirteen *H. parviporum* homokaryotic isolates out of thirty initially crossed in our previous study (Clergeot et al., 2019), and from 73 pairs of their heterokaryotic offspring were analyzed for mitonuclear epistasis in this study. Heterokaryons of each pair are carrying the same parental haploid nuclei, but mitochondria from either of the two parental isolates (Xu and Wang, 2015; see also **Figure 2** in Clergeot et al., 2019). All parental homokaryotic isolates were given a numeric code (crossing chart and correspondence of numeric codes to original names are available in Supplementary Material 1) and each isolate was labeled as follows: Homokaryotic parents are designed by their numeric code with prefix “Ho.” Heterokaryons are designed by numeric codes of their parental nuclei, starting by acceptor isolate bringing the parental cytoplasm (maternal isolate), followed by donor isolate in parenthesis (paternal isolate). For example, heterokaryon 9(32) has haploid nucleus and mitochondria of homokaryotic parent Ho9, but only haploid nucleus of homokaryotic parent Ho32. Pairs of heterokaryons originating from the same homokaryotic parents are labeled with parental numeric codes in parenthesis separated by a semicolon. For example, pair (9;32) consists of heterokaryons 9(32) and 32(9), which originate from homokaryons Ho9 and Ho32.

### Phenotypic Analysis

All data of fungal mycelium growth used in this study have been published earlier in Clergeot et al. (2019). Briefly, isolates were grown on artificial medium cast in Petri dishes, and their growth was recorded daily on two or three plates, 4–6 days post inoculation, four times on each plate. Experiments were repeated at that time, although never simultaneously with all heterokaryons together for obvious practical reasons. Consequently an “assay” does not refer to a set of experiments carried out with all isolates together at the same time, but rather to a specific experiment in a series made for each individual isolate at different times between 2015 and 2017, usually two or three. Each assay for any of the 146 heterokaryons considered in this study was given a different identification number. One heterokaryon was assayed just once, 95 twice, 41 thrice, eight four times, and one six times, in a total of 352 assays, consisting in a total of 910 different plates with four values of growth rate each.

All values of radial growth rate (RGR, in mm/day) were estimated by the method of the least squares, using published data of growth measurements of parental homokaryons and their heterokaryotic offspring (see Supplementary Material 2 gathering all growth rate data). Data dispersion was evaluated at three levels of subdivision, by comparing quartile coefficients of dispersion [QCD = (Q3-Q1)/Q3+Q1 with Q1 being the first quartile and Q3 the third quartile]. QCDs were calculated with growth rates from either each plate (910 sets of values), each assay (352 sets), or each heterokaryon (146 sets). Statistical homogeneity between data sets (i.e., heterokaryons of a same pair, or different assays of the same heterokaryon) was assessed using Mann-Whitney tests for two independent samples (two tails; α = 0.05) with correction for ties, included in the Real Statistics Resource Pack software (Release 3.5.3). Copyright (2013–2021) Charles Zaiontz. www.real-statistics.com. Risk α for Type I error was adjusted for multiple testing using the method of Holm-Bonferroni (Holm, 1979).

### Genotypic Analysis

#### Filtering of Mitochondrial SNPs

*H. parviporum* mitochondrial DNA sequence is borne by two specific unitigs, unitig_36 and unitig_37 (Clergeot et al., 2019). All SNPs present on these two unitigs were filtered as described (Clergeot et al., 2019), but using in-house Perl scripts revisited in order to include indels and to lift constraint on the quality score. All Perl scripts used in this study are available on GitHub under repository CytN (https://github.com/phcler/CytN). Positions of mitochondrial SNP loci were searched in *H. irregulare* annotated mitochondrial genome (accession NC_024555.1) using BLASTN software (https://blast.ncbi.nlm.nih.gov/Blast.cgi) and published mitochondrial ORF predictions (Himmelstrand et al., 2014).

#### Filtering of Nuclear SNPs

Nuclear SNPs were filtered based on genotypic information that one allele is borne by homokaryotic isolates Ho9 and Ho32, the other borne by Ho2, Ho5, Ho11, Ho18, Ho19, Ho20, Ho27, Ho26, Ho30 and Ho31, and that allele is undetermined for all other isolates. As Ho18 was the reference isolate for SNP calling (Clergeot et al., 2019), Ho9 and Ho32 are therefore bearing the alternative allele. Apart from setting this allelic filter, from including biallelic indels and from relaxing constraint on the quality score (lowered from 10,000 to 100), all other filtering steps were performed using in-house Perl scripts as previously described (Clergeot et al., 2019). An automated procedure was designed to find out whether or not filtered SNPs are: (1) localized in the open reading frame of a nuclear gene in *H. parviporum*, (2) at the origin of a non-synonymous mutation in isolates Ho9 and Ho32. In brief, the procedure relies on standalone nucleotide blast searches with *H. parviporum* genomic fragments surrounding each SNP as queries against a custom database including 13,405 open reading frames (ORF) of the related species *H. irregulare* (Olson et al., 2012). Blast results files were subsequently analyzed with a set of in-house Perl scripts to detect non-synonymous substitutions in the coding sequence of a gene. SNPs predicted to localize in an intron, or up to 800 bp upstream/downstream of the first/last exon of a *H. parviporum* gene were analyzed with separate in-house Perl scripts. Details about these procedures of SNP filtering and analysis are provided in Supplementary Materials 3, 4.

#### Expression Analysis of Mitochondrial Locus *nc-ORF5* in *H. irregulare*

Transcripts of the 936 bp-long hypothetical ORF (positions 46939-47874 in *H. irregulare* mitochondrial genome; accession NC_024555.1) published as *nc-ORF5* in Himmelstrand et al. (2014) were searched among published RNA sequencing data of *H. irregulare* strain TC32-1 (Olson et al., 2012) using magic-BLAST software (https://ncbi.github.io/magicblast/). Proteins similar to actual *nc-ORF5* translation product were searched using BLASTP software (https://blast.ncbi.nlm.nih.gov/Blast.cgi).

## Results

From the thirty homokaryotic isolates of *H. parviporum* in our previous study from 2019, reciprocal exchange of nuclei during mating was successful with thirteen of them crossed pairwise, leading to 73 pairs of heterokaryons having the same parental haploid nuclei, but mitochondria from either of the two parental isolates. In this study, we have used phenotypic data from these thirteen homokaryotic parents and their heterokaryotic offspring to investigate mitonuclear interactions.

### Analysis of Phenotypic Data

#### Exploratory Data Analysis

A global analysis of data structure was carried out before comparing phenotypes of homokaryotic and heterokaryotic isolates. It shows that heterokaryons are characterized globally by their phenotypic homogeneity in comparison to their homokaryotic parents, but can be divided in three groups: one small group of outliers with markedly lower maximal growth rate (3 isolates), a large group with high maximal growth rate and low phenotypic variance (107 isolates), and a medium group with high maximal growth rate, but a high phenotypic variance that seems intrinsic to their parental combinations (36 isolates).

##### Data Dispersion

Dispersion of growth rate data was analyzed both in the heterokaryons and their homokaryotic parents to detect those with high level of data dispersion, and, if so, to identify a plausible cause for it. Quartile coefficients of dispersion (QCD) of growth rate data were calculated at different levels of subdivision (within Petri dish, assay, or isolate). Their distributions show that differences of growth rate between assays (isolate level of data subdivision) contribute the most to data dispersion: while 0.76% of the dishes data and of 7.1% of the assays data have a QCD larger than 0.125, data of 38 heterokaryons (26 %) have a QCD larger than this value. A QCD larger than 0.125 was subsequently considered the hallmark of high level of data dispersion throughout this study. Data dispersion between assays in the parental homokaryons is weakly correlated to a lower median growth rate (*r*^2^ = 0.55714). In the heterokaryons however, a high QCD is neither explained by a low median growth rate (*r*^2^ = 0.26644), or by having one or two parents with a high QCD (*r*^2^ = 0.0155). This analysis ruled out the possibility that high level of data dispersion could have experimental cause and rather points at specific heterokaryotic combinations to explain this phenomenon.

##### Data Distribution

**Figure 1:**
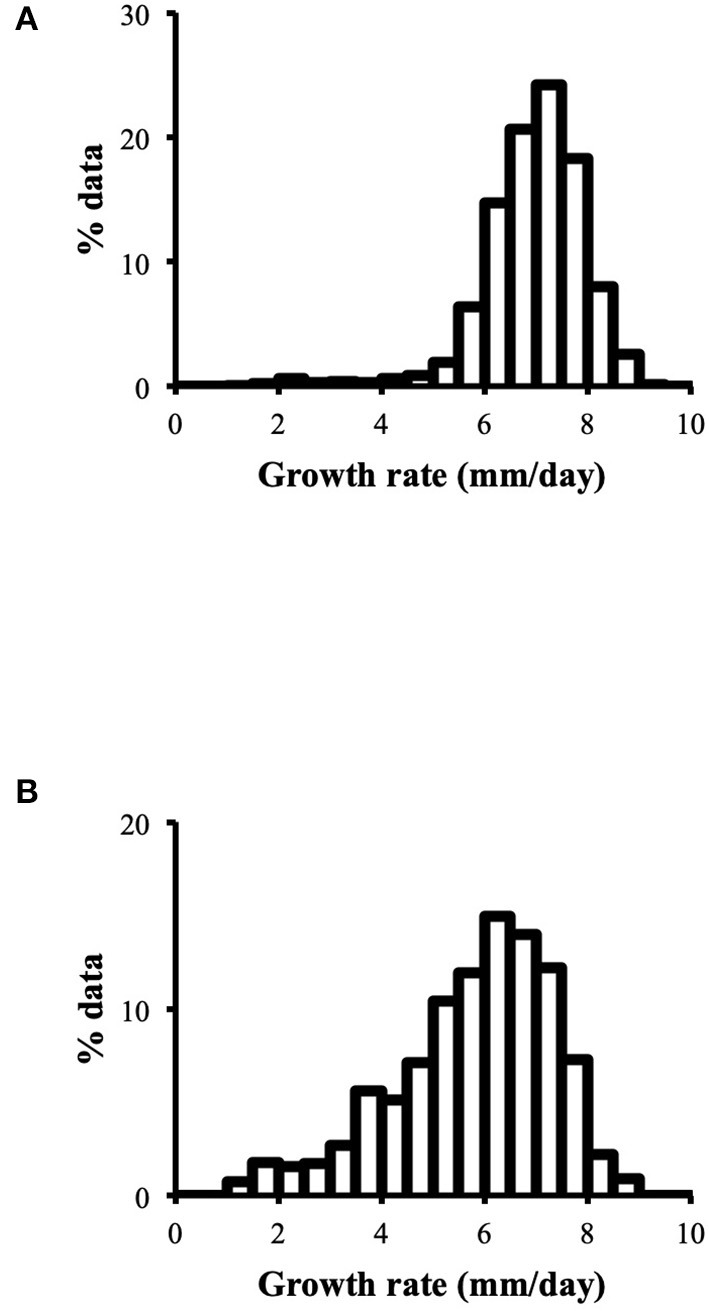
**(A)** Data distribution of growth rate assays with the highest median of each heterokaryon is narrow and symmetric (high_med; *n* = 1,563); **(B)** Data distribution of growth rate assays with the lowest median of each heterokaryon is broad and left-skewed (low_med; *n* = 1,487); binwidth = 0.5.

**Figure 2:**
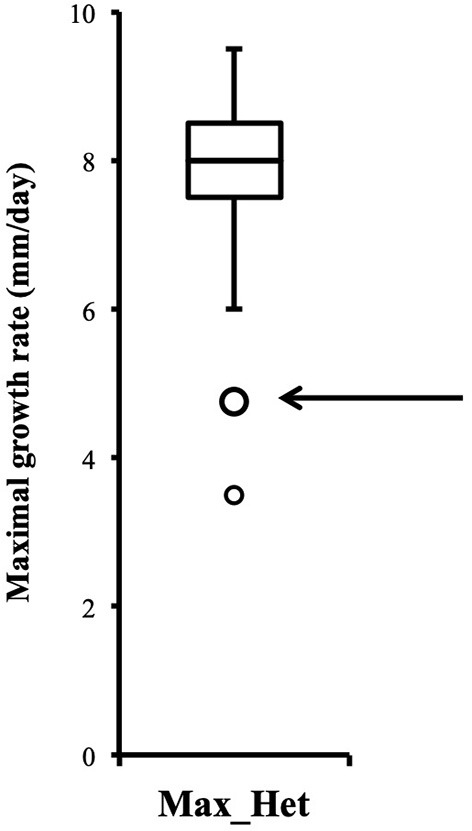
Distribution of heterokaryon maximal growth rates is narrow, symmetric, and centered on a high value (*n* = 146). Maximal growth rate of heterokaryon 9(32) is one of the two outliers indicated by the arrow.

**Figure 3:**
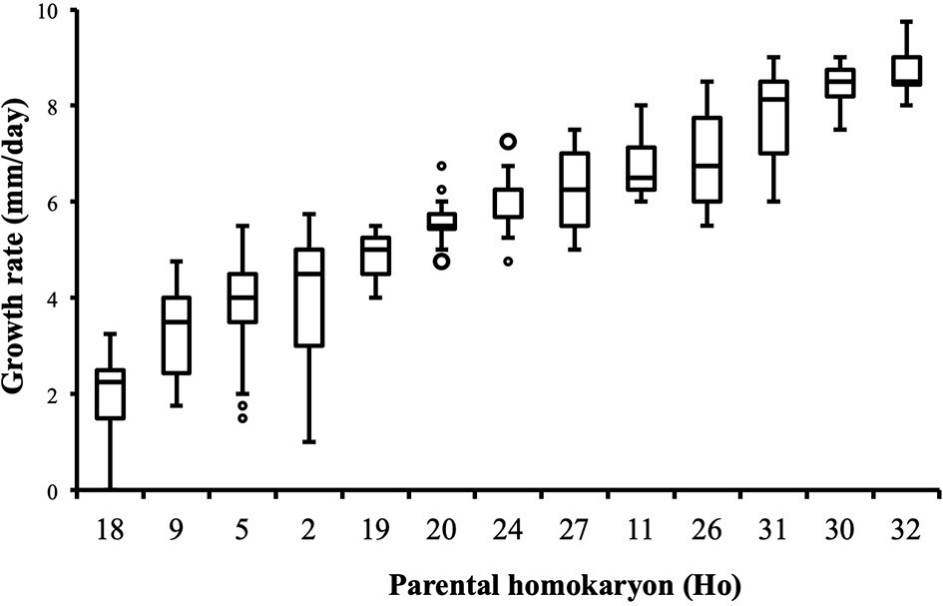
Parental homokaryons display a large array of distributions of growth rate data. Maxima of the slowest parents are below minima of the fastest; *n* = 24 (Ho20, Ho24), *n* = 48 (Ho2, Ho5), or *n* = 36 (all other isolates).

**Figure 4:**
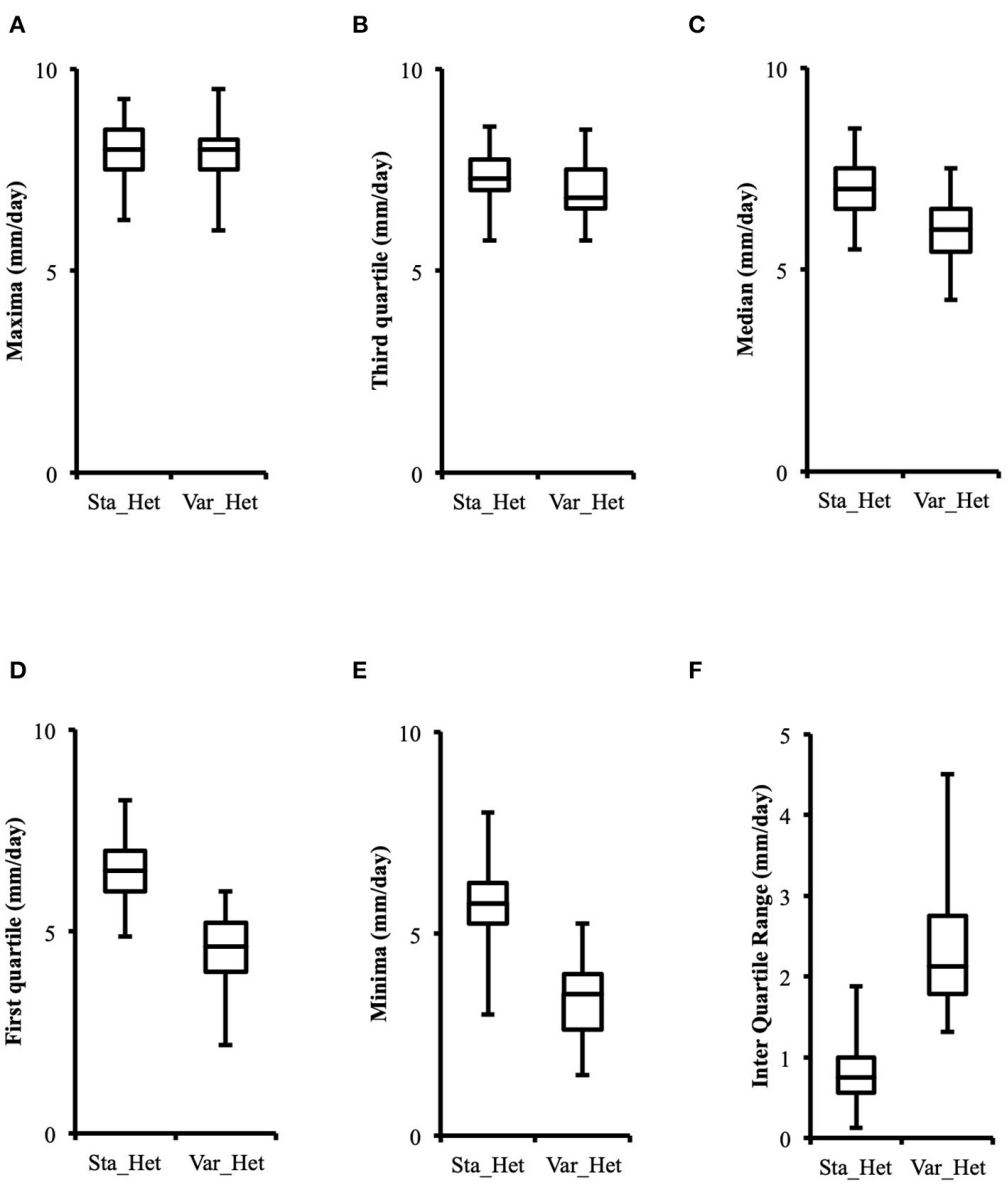
Distributions of higher values of growth rate in phenotypically stable heterokaryons (sta_het; *n* = 107) and in phenotypically variable heterokaryons (var_het; *n* = 36) are similar, but the latter have a larger data spread toward lower values. **(A)** Maxima; **(B)** Third quartile; **(C)** Median; **(D)** First quartile; **(E)** Minima; **(F)** Inter quartile range.

Distribution of growth rate data was analyzed both in the heterokaryons and in their homokaryotic parents. Similar to what was previously published for the entire cohort of isolates (see **Figure 3** in Clergeot et al., 2019), data distribution of the thirteen parents is multimodal, indicating some genetic polymorphism for growth rate, whereas it is unimodal for the 146 heterokaryons, showing a homogenization of phenotypic variance in the offspring (Supplementary Figure 1). The center of the heterokaryon data distribution is shifted toward values recorded in the fastest parents only. Data of the assay with the highest median of each heterokaryon were then regrouped in a separate subset called high_med. Similarly, data of the assays with the lowest median were regrouped in another subset called low_med. Distributions of both subsets were compared. For high_med, distribution is narrow and symmetric, centered on a high value of growth rate (median = 7.25 mm/day), with very few outliers toward the lower values (Figure 1A). For low_med, distribution is broad and left-skewed, (Figure 1B), although its center remains close to high values of growth rate (median = 6.25 mm/day). Distribution of maximal growth rate (Figure 2) shows at the same time that almost all heterokaryons have the capacity to grow at rate only found in the fastest parents (Figure 3). Out of 146 maximal growth rate values, 143 are symmetrically distributed around a median very close to 8 mm/day and have an inter-quartile range (IQR) of 1.25 mm/day (Figure 2). Three outliers in low values of growth rate (max_out) correspond to heterokaryons 18(24), 24(18), and 9(32) and have maxima ≤ 4.75 mm/day. The 143 heterokaryons with high maxima (excluding the three outliers) were divided in two groups according to their QCDs: one group of 107 heterokaryons with a low data dispersion (QCD ≤ 0.125; subset named sta_het), another group of 36 heterokaryons with higher data dispersion (QCD > 0.125; subset named var_het). Distributions of their maxima, third quartiles, medians, first quartiles, minima, and IQR were then compared (Figure 4). For a quarter of the heterokaryons, growth rate varies more between assays, but their maxima and third quartiles are similar to the ones of the 75% majority (Figures 4A,B). Spread and left-skewness of the data distribution of all heterokaryons is therefore rather explained by the larger phenotypic variance between assays recorded in these 36 isolates—shifting their median growth rate toward lower values (Figures 4C–E) and increasing their IQR (Figure 4F)—than by intrinsic difference of growth rate, observed for example between the slowest and the fastest parents (Figure 3).

#### Statistical Analyses of Phenotypic Data

**Figure 5:**
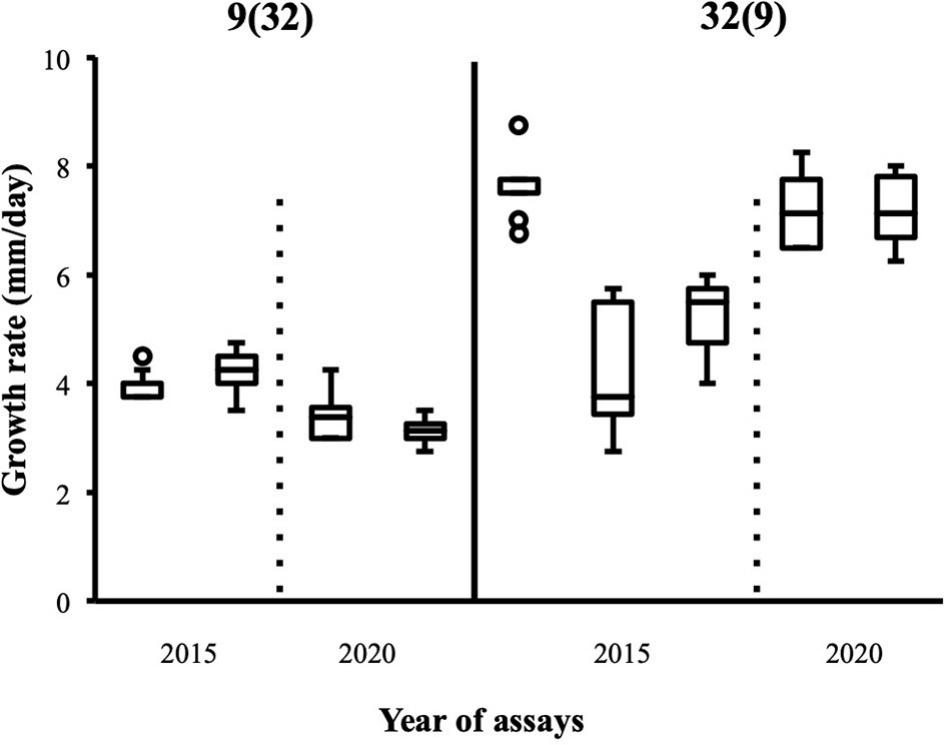
Replicates of growth rate assays carried out with heterokaryon pair (9;32) confirm original phenotypes deduced from assays with the highest median; 2015: data published in Clergeot et al. (2019): data from this study (*n* = 24).

**Figure 6:**
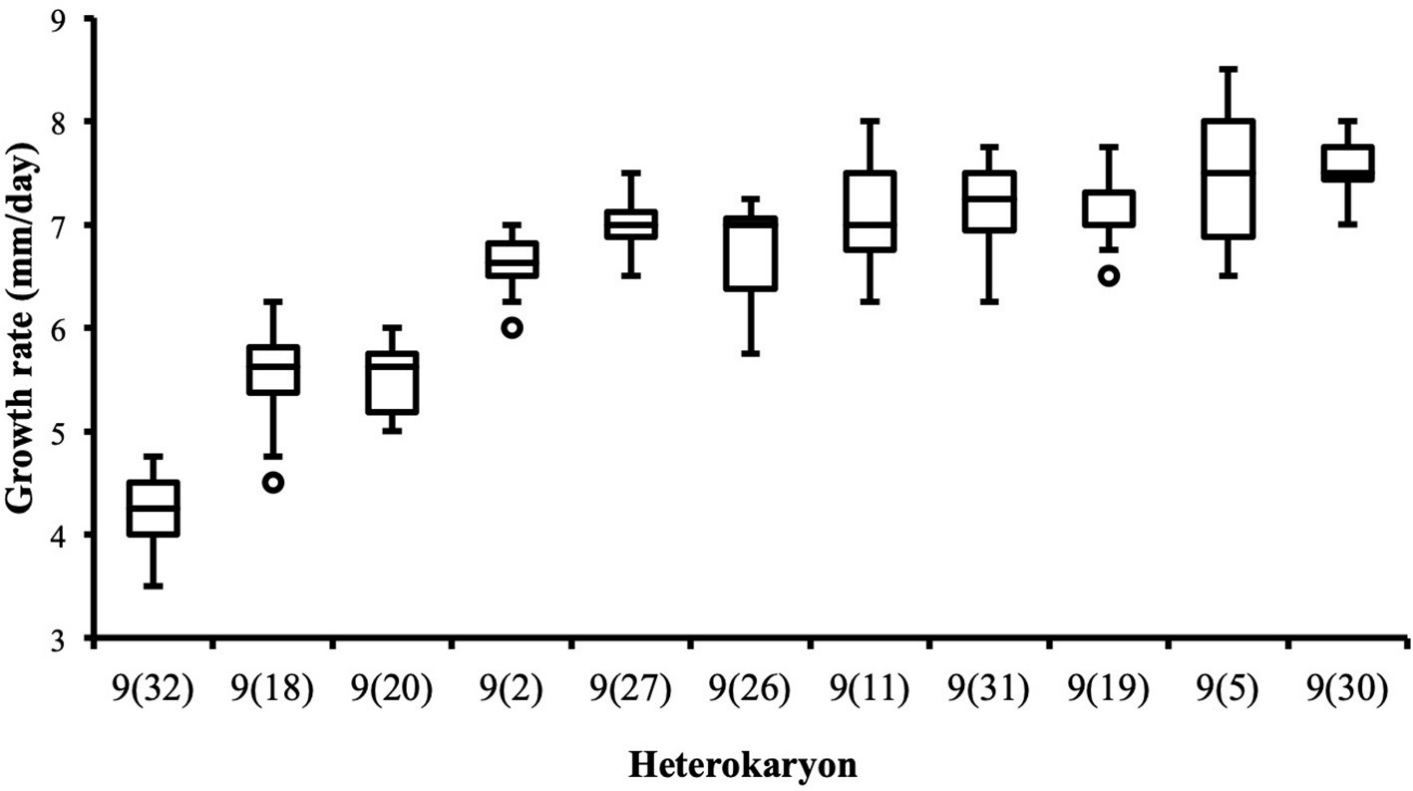
Heterokaryon 9(32) grows significantly slower than any other heterokaryon made with Ho9 as acceptor parent. 2-tails Mann-Whitney tests were performed with data of the assays with the highest median for each isolate. Risk α for type I error was adjusted for multiple testing (*n* = 10; initial value = 0.05).

Although the 143 heterokaryons with high maximal growth rate have similar genetic potential for this trait, their difference of phenotypic variance could bias statistical analyses based on their median growth rate. Therefore, two series of statistical analyses were performed with the 73 pairs of heterokaryons, first using all data, then using high_med data subset (complete results of the tests in Supplementary Material 5, tabs “MW tests—all data” and “MW tests—highest median”). Six pairs show a statistically significant difference of median growth rate in both series of tests (see Table 1). For heterokaryon pair (9;32), this support is the highest of all pairs using high_med. With this data subset, pair (9;32) has the largest difference of median growth rate of all pairs (3.5 mm/day), far above any other (next is (24;32) with 1,375 mm/day). Moreover, heterokaryon 9(32) displayed a low growth rate in two independent assays, something that only two other heterokaryons did, 18(24) and 24(18). Its maximal growth rate is a low outlier in the distribution of maxima of all heterokaryons (Figure 2). A complementary phenotypic analysis of pair (9;32) was carried out and compared with the one published in 2019 for the same pair (Figure 5). Repeated growth rate experiments with heterokaryons 9(32) and 32(9) confirmed their difference of phenotype [growth rate data from 2020 Figure 5; Mann-Whitney *U* = 0; n1 = n2 = 24; median_9(32)_2_ = 3,25 mm/day; median_32(9)_2_ = 7,125 mm/day; effect size *r* = 0.8589; *P* < 0.001; two tailed]. This data subset was used to show that 9(32) has indeed the slowest growth rate of all heterokaryons formed with Ho9 as acceptor parent, pointing out the fact that its combination with Ho32 is unique in this sample (Figure 6; complete results of statistical tests in Supplementary Material 5, tab “MW tests – Acc9”).

**Table 1:** Pairs of heterokaryons with statistically different median growth rates; _het: all data of the heterokaryon were used; _high: data of the assay with the highest median of each heterokaryon were used; QCD: quartile coefficient of dispersion; Med: median growth rate; *p: p*-values of 2-tails Mann-Whitney tests; α: risk for type I error adjusted for multiple testing (*n* = 73; initial value = 0.05).

	**Heterokaryon pair**					
	**(9;32)**	**(30;32)**	**(2;11)**	**(9;18)**	**(18;32)**	**(24;32)**
het1	9(32)	30(32)	11(2)	9(18)	32(18)	32(24)
het2	32(9)	32(30)	2(11)	18(9)	18(32)	24(32)
QCD_het1	0.0698	0.1256	0.0890	0.0279	0.1415	0.0894
QCD_het2	0.2435	0.0625	0.0404	0.0660	0.2821	0.1351
Med_het1	4	6.25	6	5.5	6.5	7.5
Med_het2	5.625	7.75	6.75	6	5.5	5.875
*p*	0.00035	2.42E-06	5.27E-05	0.00076	7.67E-05	3.41E-08
α	0.00079	0.00069	0.00074	0.00081	0.00075	0.00068
QCD_high1	0.0588	0.0373	0.0400	0.0391	0.0407	0.0192
QCD_high2	0.0164	0.0186	0.0357	0.0467	0.0291	0.0446
Med_high1	4.25	7.625	6.125	5.625	7.75	8.125
Med_high2	7.75	8.25	6.75	6.625	6.5	6.75
*p*	3.03E-05	8.75E-05	0.00011	0.00013	0.00044	0.00075
α	0.00068	0.00069	0.00070	0.00071	0.00074	0.00076

Subsequent analysis focused on three specific pairs, (9;32), (18;32), and (9;18), for three reasons: (1) differences of phenotype of their heterokaryons are statistically grounded; (2) the complete offspring resulting from reciprocal crossings of their three parents is available for a grouped analysis; (3) based on results of previous SNP call and filtering, mitochondrial haplotypes of parents Ho9 and Ho18 were supposed to be identical, which was expected to facilitate genotyping. Using data of the assay with the highest median of each heterokaryon and each parent of these three pairs, a series of 36 statistical pairwise tests was carried out, in order to assess differences of growth rate (complete results of the tests in Supplementary Material 5, tab “MW tests – 3 pairs”). Difference of median growth rate is significant for all combinations, except 32(9)-32(18), 18(9)-18(32), and 9(32)-Ho9 (Table 2).

**Table 2:** *p*-values of statistical tests performed to compare growth rates of heterokaryons from pairs (9;32), (9;18), and (18;32) and their parents Ho9, Ho18, and Ho32.

	**9(32)**	**32(9)**	**18(32)**	**32(18)**	**9(18)**	**18(9)**	**Ho9**	**Ho18**	**Ho32**
9(32)		3.03E-05	2.99E-05	3.12E-05	0.00015	3.08E-05	0.6821	2.88E-05	2.91E-05
32(9)	3.03E-05		0.00016	0.66174	3.05E-05	0.00029	2.97E-05	2.81E-05	9.70E-05
18(32)	2.99E-05	0.00016		0.00044	1.79E-04	0.64118	2.93E-05	2.78E-05	2.80E-05
32(18)	3.12E-05	0.66174	0.00044		3.15E-05	0.00107	3.06E-05	2.91E-05	2.93E-05
9(18)	0.00015	3.05E-05	0.00018	3.15E-05		0.00013	0.00013	2.91E-05	2.93E-05
18(9)	3.08E-05	0.00029	0.64118	0.00107	0.00013		3.01E-05	2.86E-05	2.88E-05
Ho9	0.68209	2.97E-05	2.93E-05	3.06E-05	0.00013	3.01E-05		2.83E-05	2.85E-05
Ho18	2.88E-05	2.81E-05	2.78E-05	2.91E-05	2.91E-05	2.86E-05	2.83E-05		2.70E-05
Ho32	2.91E-05	9.70E-05	2.80E-05	2.93E-05	2.93E-05	2.88E-05	2.85E-05	2.70E-05	

*2-tails Mann-Whitney tests were performed with data of the assay with the highest median for each isolate; risk α for type I error was adjusted for multiple testing (n = 36; initial value = 0.05). For the combinations shaded in blue, the hypothesis of an identical data distribution could not be rejected*.

### Genotypic Analysis

**Figure 7:**
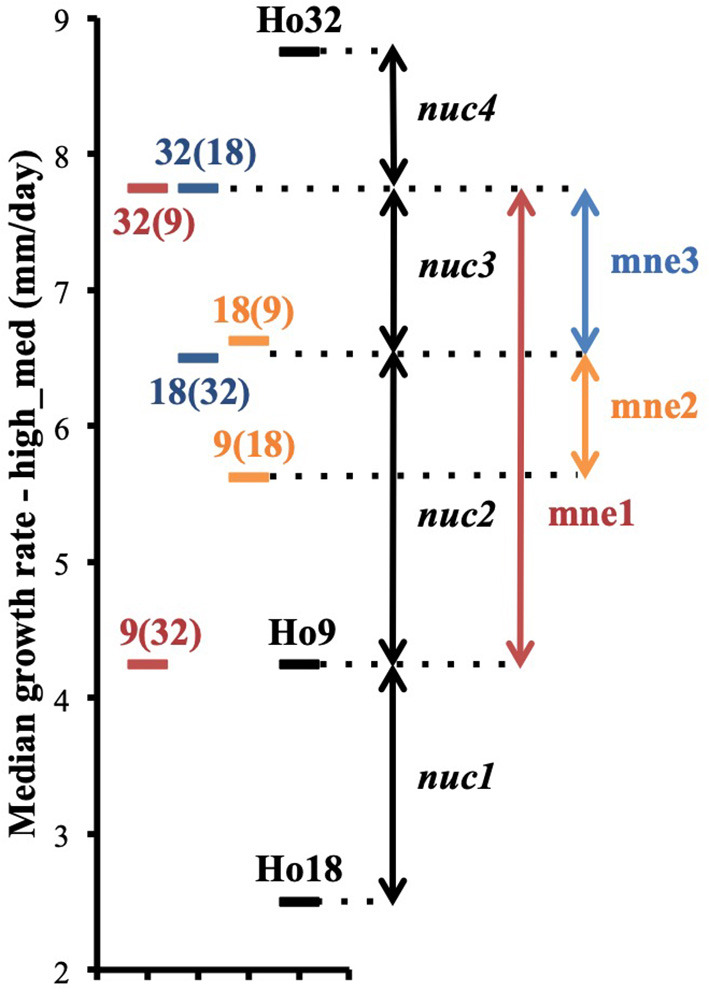
Phenotypic differences between heterokaryons from pairs (9;32), (9;18), (18;32) and their parents are explained by the contribution of four different nuclear genetic loci *nuc1, nuc2, nuc3*, and *nuc4* and the intervention of three events of mitonuclear epistasis mne1, mne2, and mne3 (phenotypes are based on data of the assay with the highest median for each isolate; median growth rates are plotted on the figure).

Phenotypic variance between the six isolates of pairs (9;32), (18;32), (9;18), and their three parents are best explained by the intervention of different alleles at a minimum of four nuclear loci, *nuc1, nuc2, nuc3*, and *nuc4*, and by non-additive variance due to three events of mitonuclear epistasis named mne1, mne2, and mne3 (Figure 7). Specific efforts were made to characterize *nuc2*, but also mne1 and mne2, as both presumably involve *nuc2*. The fact that heterokaryon 9(32) grows significantly slower than any other heterokaryon made with Ho9 as acceptor parent (Figure 6) shows that Ho9 and Ho32 are the only homokaryotic isolates to carry a recessive allele of *nuc2*. This allele, *nuc2-1*, interacts negatively with an allele of mitochondrial locus *mt2* borne by Ho9 only. Based on previous SNP calling and filtering, Ho9 and Ho18 were however supposed to have the same mitochondrial haplotype, which contradicts the fact that difference of growth rate between 9(18) and 18(9) is significant too. Other filtering of the SNPs called from nuclear and mitochondrial genome sequences of all homokaryotic isolates were set in order to identify markers linked to these two loci.

#### Mitochondrial Locus *mt2*

**Figure 8:**
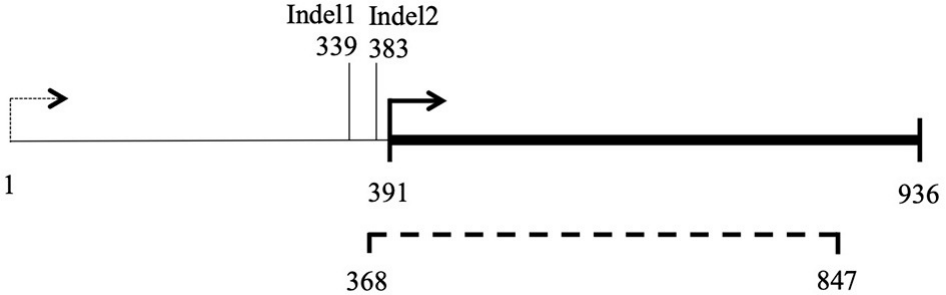
Map of locus *nc-ORF5*. 1–936 bp: ORF prediction in *H. irregulare* mitochondrial genome annotation; Dashed line (368–847 bp): transcription detected in *H. irregulare* isolate TC32-1; Thick line (391–936 bp): actual *nc-ORF5*.

Filtering of mitochondrial SNPs was revisited and performed in a way that include indels. This led to the identification of three indels whose alleles are different in Ho9 and Ho18 (see complete list of 216 SNPs resulting from this filtering in Supplementary Material 6, tab “Mitochondrial SNPs”). Two were found in non-coding DNA, but one is localized in a non-conserved mitochondrial locus homologous to *nc-ORF5* in the mitochondrial genome sequence of *H. irregulare* (TAA/TAAA in position 472 of unitig_37; ORF is on the minus strand of this unitig). In *H. irregulare, nc-ORF5* is predicted to encode a protein of unknown function and made of 311 amino acid residues, annotated in the mitochondrial proteome of *H. irregulare* under NCBI accession YP009048485.1. Transcript and coding sequence of this locus were found to be shorter than predicted in the 5' end (181 aa instead of 311 in *H. irregulare*; see Figure 8A and magic-blast output in Supplementary Material 7), which is in agreement with the results of a protein similarity search showing that, although hypothetical orthologs of this protein exist in other Russulales fungi, no similar proteins are found in other species except for a hypothetical one in soilborne gammaproteobacteria with a relatively high sequence similarity and a similar length to nc-ORF5p (query length: 181 aa; subject length: 194 aa; query cover: 83%; 58% identity; E = 3e-51; subject GenBank ID: TLY78166.1). Consequently, it is reasonable to assume that this *H. parviporum* indel is not located within *nc-ORF5* itself, but either in its promoter, or its 5' untranslated transcript sequence (5' UTR). Interestingly, a second indel identified after mitochondrial SNP filtering is localized 44 bp downstream of the first SNP, in the promoter/5' UTR sequence of the same ORF (TAA/TAAA in position 428 of unitig_37; ORF is on the minus strand of this unitig). Ho9 and Ho18 have the same allele of this indel. Overall, three different alleles of *nc-ORF5* promoter/5' UTR exist in *H. parviporum* homokaryons used in this study, depending on the allelic combination of these two indels: Ho18 and Ho32 have a first allele *mt2-1*, Ho9, Ho11, Ho20, Ho26, Ho27, Ho30, and Ho32 have a second allele *mt2-2*, and Ho2, Ho5, and Ho24 have a third one *mt2-3* (Table 3). In the specific context of the crossings of Ho9, Ho18, and Ho32, it is worth noticing that Ho18 and Ho32 have the same allele, *mt2-1*, while Ho9 has allele *mt2-2*.

**Table 3:** Mitochondrial locus *mt2* exists in three different forms, *mt2-1, mt2-2*, and *mt2-3*, in the thirteen *H. parviporum* parental homokaryons depending on their indels alleles.

**Homokaryotic isolate (Ho)**	**Indel1 allele**	**Indel2 allele**	**Locus allele**
18; 32	TAA	TAA	*mt2-1*
9; 11; 19; 20; 26; 27; 30; 31	TAAA	TAA	*mt2-2*
2; 5; 24	TAAA	TAAA	*mt2-3*

The best way to explain the mitonuclear epistasis events observed (Table 4) is to invoke:

**Table 4:** Expression of nuclear locus *nuc2* depends on appropriate allelic combination with mitochondrial locus *mt2*; E: full expression of the trait; e: partial expression; –: no expression; *nuc2* (d): *nuc2* allele brought by the donor isolate in heterokaryons.

	***nuc2-1***	***nuc2-2***
***mt2-1***	E	-
***mt2-2***	–	e
	**Ho9**	**Ho18**	**Ho32**	**9(18)**	**18(9)**	**9(32)**	**32(9)**	**18(32)**	**32(18)**
*nuc2*	*nuc2-1*	*nuc2-2*	*nuc2-1*	*nuc2-1*	*nuc2-2*	*nuc2-1*	*nuc2-1*	*nuc2-2*	*nuc2-1*
*nuc2* (d)				*nuc2-2*	*nuc2-1*	*nuc2-1*	*nuc2-1*	*nuc2-1*	*nuc2-2*
*mt2*	*mt2-2*	*mt2-1*	*mt2-1*	*mt2-2*	*mt2-1*	*mt2-2*	*mt2-1*	*mt2-1*	*mt2-1*
Expression	–	–	E	e	E	–	E	E	E

- for mne1, a negative interaction either between mitochondrial allele *mt2-2* and nuclear allele *nuc2-1* borne by Ho9 and Ho32 but not Ho18, or between mitochondrial allele *mt2-1* and nuclear allele *nuc2-2* borne by Ho18;
- for mne2, a partially negative interaction between mitochondrial allele *mt2-2* and nuclear allele *nuc2-2*, as observed in heterokaryon 9(18).

#### Nuclear Locus *nuc2*

**Figure 9:**
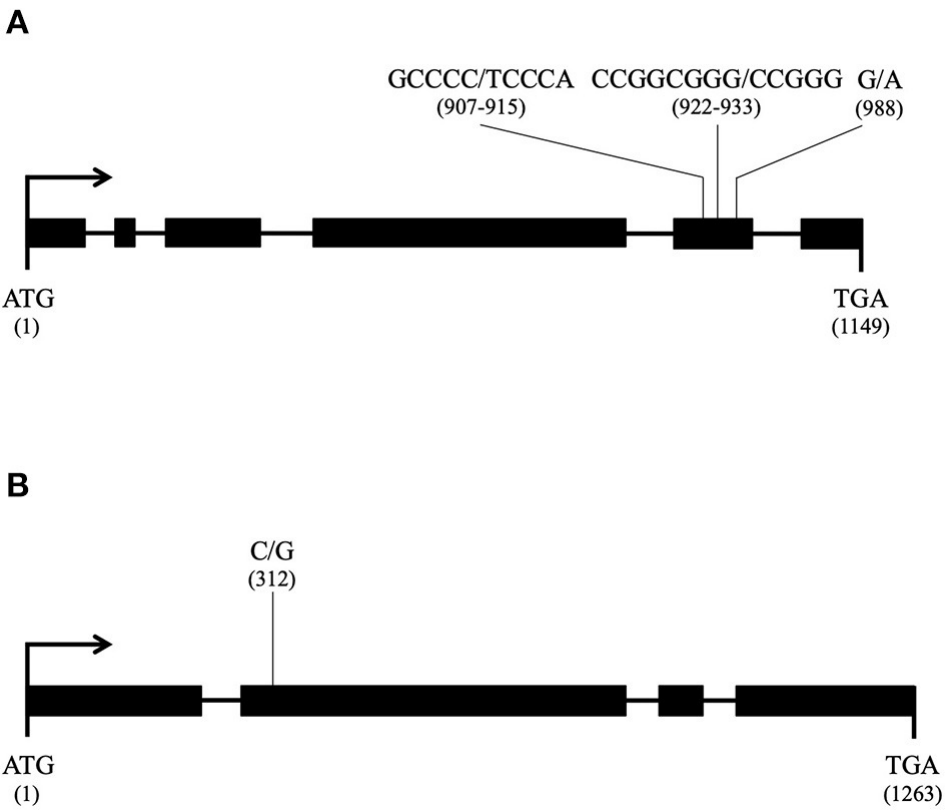
Candidate nuclear loci for *nuc2* in *Heterobasidion parviporum* and their mutation profile (thick line: exon; thin line: intron; for each mutation, the reference allele is given first and its position in the coding sequence is in parentheses); **(A)** Gene encoding a mitochondrial carrier protein orthologous to *Heterobasidion irregulare* XM_009546414.1; **(B)** Gene encoding a mitochondrial calcium uniporter orthologous to *Heterobasidion irregulare* XM_009552795.1.

From the phenotypes of all other heterokaryons obtained with parent Ho9 as nuclear acceptor, it was possible to deduce that homokaryotic isolates Ho2, Ho5, Ho11, Ho19, Ho20, Ho27, Ho26, Ho30, and Ho31 cannot bear recessive allele *nuc2-1* for sure (Figure 6). This information was used to set an allele-based filter for nuclear SNPs in order to provide candidate loci for *nuc2*, which led to the isolation of 1,242 SNPs. DNA of these SNP loci were first compared with a database made of all *H. irregulare* ORFs. For each locus matching an ORF, the SNP was localized in *H. parviporum* genomic DNA in order to identify those whose alternative allele causes a non-synonymous mutation. 449 SNPs were confirmed to be in an *H. parviporum* exon, of which 158 are responsible of a non-synonymous mutation in 78 different ORFs. Finally, the annotation of their *H. irregulare* orthologs were checked to select those associated with the mitochondria, either by localization or by function. This process of successive filtering led to the identification of two candidates for *nuc2* [see details in Supplementary Material 6, tab “Nuclear SNPs (non-syn)”]:

- A gene encoding a mitochondrial carrier protein (*H. irregulare* accession number: XM_009546414.1) with the following mutation events, all in exon 5 (Figure 9A): two consecutive nucleotide transversions (G to T and C to A) leading to an amino acid substitution G to V in position 303, the deletion of three consecutive nucleotides GCG leading to the deletion of amino acid A in position 309 in Ho7, Ho9, Ho12, Ho15, Ho32, and Ho35, and a nucleotide transversion G to A leading to amino-acid substitution R to K in position 330 in isolates Ho9 and Ho32;
- A gene encoding a hypothetical mitochondrial calcium uniporter (*H. irregulare* accession number: XM_009552795.1) with a single mutation due to a nucleotide transition C to G in exon 2 (Figure 9B) leading to amino acid substitution I to M in position 104 in Ho9, Ho23, Ho25, and Ho32.

Of the 793 SNPs not localized into an exon of a *H. parviporum* gene whose ortholog is transcribed in *H. irregulare*, 130 were found into an intron of such a gene, 86 were found <800 bp upstream of the first exon, and 68 <800 bp downstream of the last exon. Annotations of these genes were checked for possible association with the mitochondria, leading to the isolation of eight SNPs of interest [see details in Supplementary Material 6, tab “Nuclear SNPs (All)”]. Five SNPs were found into two introns of the gene encoding the mitochondrial carrier already mentioned (*H. irregulare* accession number: XM_009546414.1). One SNP was found 60 bp downstream of the stop codon of the gene encoding the hypothetical calcium uniporter already mentioned (*H. irregulare* accession number: XM_009552795.1). One SNP was found 113 bp upstream of the start codon of a gene encoding a beta subunit a of putative F1F0-type ATPase (*H. irregulare* accession number: XM_009544597.1). One SNP was found 11 bp upstream of the start codon of a gene encoding another mitochondrial carrier protein (*H. irregulare* accession number: XM_009545544.1).

## Discussion

Our phenotypic data show that two major competing trends are at work in heterokaryons, with opposite effects on growth rate. The first trend is “heterokaryon vigor” (Clergeot et al., 2019), which results from the reciprocal masking of deleterious alleles brought by one or both parents (Clark and Anderson, 2004). Our growth rate data show that dominance is frequent, but overdominance is rare and limited to the offspring of parents that are both less fit. As the fittest parental alleles are overwhelmingly dominant, the center of the distribution of all heterokaryons data is shifted toward the position it would have in a hypothetic homokaryon having the largest combination of the fittest parental alleles. Homokaryons growth rate data suggest that the fastest parents in our sample are close to such an optimal allelic combination, as none of the heterokaryotic offspring can grow at a higher rate than they do.

The second trend is high heterokaryon-specific phenotypic variation, a phenomenon that we have observed in several heterokaryons (26%) after carrying out different growth rate assays over time. These heterokaryons can nevertheless grow as fast as the others. Their genetic potential for this specific trait is therefore not different from the rest of the heterokaryons. The fact that their phenotype does vary more between assays is due to other factors, unidentified so far, although probably linked to the heterokaryotic state, during which parental haploid nuclei coexist in the same cytoplasm, but do not fuse (Raper, 1966). The heterokaryon is an intermediary state between haploidy and true diploidy, more plastic than the latter due to the possibilities of remating (Hansen et al., 1993; Johannesson and Stenlid, 2004; James et al., 2009), secondary formation of haploid sectors (Stenlid and Rayner, 1991; Hansen et al., 1993), and production of haploid conidia (Hansen et al., 1993). Although considered as functionally equivalent to a diploid (Day and Roberts, 1969), such a dynamic and possibly conflicting association of haploid nuclei is likely to lead to stochastic variation of gene expression (Kilfoil et al., 2009), and therefore, of heterokaryon phenotype. Degree of phenotypic variation of each heterokaryon might be genetically determined by specific combination of parental alleles, as for nuclear ratio in heterokaryons of *H. parviporum* (James et al., 2008).

Analysis of heterokaryons growth rate data showed that higher values reflect their genetic potential for this trait better, which made possible the identification of pairs of heterokaryons issued from the same parents, but whose phenotypes are different. It confirms our previous detection of mitochondrial genetic effects in the same cohort of heterokaryons of *H. parviporum* (Clergeot et al., 2019). In one occurrence, owing to the facts that two non-kin-related parental isolates (Ho9 and Ho18) have nearly identical mitochondrial haplotypes and that their crossings with a third isolate, Ho32, gave heterokaryons with clear-cut phenotypes, we have identified alleles of a mitochondrial locus (*mt2*) and a genomic locus (*nuc2*) involved in a genetic interaction in *trans* across the two genomes. Different combination of alleles at these two loci contribute to phenotypic variation in the offspring of the crossings with these three isolates.

Candidates for genomic locus *nuc2* were searched using phenotypic information of heterokaryons from all other crossings involving parent Ho9 and SNPs markers called from the genome sequences of all homokaryotic isolates (Clergeot et al., 2019). In this work, SNP alleles associated with a non-synonymous mutation in two *H. parviporum* ORFs were investigated in priority as more likely candidates for *nuc2*, although other causes of phenotypic variation cannot be excluded at this stage. Similar bioinformatics methods have been used to track polymorphism in gene regulatory sequences or introns too, and led to the identification of two additional candidates for *nuc2*. The two genes whose sequences in Ho9 and Ho32 are bearing mutations altering the structure of their translation products (although it cannot be ascertained that the function of these products is modified), but also carrying mutations that might affect their expression level are considered the best possible candidates at this stage. Both are involved in molecular trafficking to the mitochondrion (a carrier protein and a calcium uniporter). Although the gene encoding a calcium uniporter cannot be ruled out at this stage, the other one encoding a mitochondrial carrier protein is a more likely candidate for *nuc2* for several reasons: Mutations in this gene result from multiple transversions (3) and one indel in the same exon, whereas the mutation in the gene encoding the calcium uniporter results from a single transition; A multiple protein alignment with orthologs from other species shows that substitution R to K in the sequence of the carrier is in a conserved motif, and this substitution is present in Ho9 and Ho32 only; Amino-acid substitutions/deletion observed in the carrier are more likely to lead to a change of balance between structure and function.

In contrast, it is not a mitochondrial gene encoding a key protein in oxidative metabolism that has been uncovered as the potential candidate involved in a mitonuclear interaction with *nuc2*, but a non-conserved gene instead, with orthologs in the Russulales order only. The ortholog of this gene in the mitochondrial genome of *H. irregulare* has been previously identified as *nc-ORF5* (non-conserved Open Reading Frame 5; Himmelstrand et al., 2014). The presence of nc-genes in mitochondrial DNA is a typical feature of Agaricomycetes fungi. Nc-genes are supposed to share the same evolutionary origin as the mitochondrial plasmid DNA frequently found in the mitochondrial genome of species in this class of fungi and contribute to explain its unusually large size. This non-conserved and exchangeable part of Agaricomycetes mitochondrial DNA might originate from the uptake of plasmids by mitochondria and its subsequent integration into mitochondrial DNA early in the evolution of this class of fungi (Himmelstrand et al., 2014; Medina et al., 2020). Protein blast searches made with the translation of the ortholog of *nc-ORF5* in *H. parviporum* (Hparv-nc-ORF5p) and by excluding proteins from the Russulales retrieved exclusively a hypothetical protein from soilborne gammaproteobacteria (Diamond et al., 2019). Phenotypic variation associated with mitochondrial DNA sequence polyporphism at this locus seems to originate from a change in the regulation of *nc-ORF5* expression rather than from its loss of function, as the SNP is located upstream of *nc-ORF5* coding sequence, either in its promoter or its 5' UTR sequence.

Further investigation would imply (1) studying the expression of all mitochondrial and nuclear candidate genes to confirm or infirm hypotheses made about them, (2) providing molecular genetics evidence of mitonuclear interaction by carrying complementation experiments of homokaryon Ho9 with wild-type copies of candidate nuclear loci for *nuc2*. Genetic transformation of *Heterobasidion spp*. using *Agrobacterium tumefaciens* has been described previously (Samils et al., 2006), including in *H. parviporum* (Ihrmark, *unpubl*.), and could be used for that purpose. Another option would be to shuffle mitochondria into various homokaryotic nuclear backgrounds by reisolating homokaryotic conidia from heterokaryons cultures (Ramsdale and Rayner, 1994) and assessing their phenotype. Finally, although our phenotypic analysis points at a very simple genetic explanation for the phenotypic variation observed in this sample of isolates (homogeneization of the heterokaryons phenotypes to the level observed in the best homokaryotic parents is the signature of the complementation of a few recessive and defective alleles unique to each slower parent by dominant wild-type alleles borne by any other parent), mitonuclear interactions involving more than one locus in the mitochondrial and/or the nuclear genome cannot be excluded so far. It could be determined by carrying out a segregation analysis (Lind et al., 2005).

Mitonuclear interactions are suspected to be the cause of some breakthrough increase of virulence or change of host range in rare interspecific hybrids (Olson and Stenlid, 2001; Giordano et al., 2018; Hu et al., 2020), but they are also part of the ordinary evolutionary process of each fungal pathogenic species and could hence contribute to mold the interactions with its host. Coadaptation between mitochondrial and nuclear DNA might be an important driver in the evolution of host-pathogen interactions. Several examples have been documented in other fungal pathogens of a change of host range or of a variation in virulence associated either with polymorphism of mitochondrial DNA, with a loss of function, or with a differential expression of nuclear genes encoding mitochondrial proteins (Monteiro-Vitorello et al., 1995; Lorenz and Fink, 2001; Inoue et al., 2002; Zhan et al., 2004; Ma et al., 2009; Mahlert et al., 2009; Patkar et al., 2012; Zhang et al., 2012; Khan et al., 2015; van de Vossenberg et al., 2018). In the context of this work, it is worth noticing that a nuclear-encoded mitochondrial carrier protein has already been mentioned in *Heterobasidion* spp. as possibly involved in virulence variation related to mitochondrial background (Giordano et al., 2018; Hu et al., 2020). Furthermore, in pathogenic Basidiomycetes fungi like *Heterobasidion spp*., the capacity of heterokaryon remating (Johannesson and Stenlid, 2004) gives mitonuclear interactions the opportunity to add to the selection process for fitter heterokaryotic associations while genetically diverse heterokaryons grow, meet and exchange nuclei on host wood. Our work brings forth evidence that the investigation of mitonuclear interactions is possible for a trait like hyphal growth rate, but the same could be considered for virulence.

## Data Availability Statement

The datasets presented in this study can be found in online repositories. The names of the repository/repositories and accession number(s) can be found in the article/Supplementary Material.

## Author Contributions

P-HC and ÅO: conceived and designed the experiments, performed the experiments, analyzed the data, and revised the paper. P-HC: exploratory data analysis, statistics, and bioinformatics and wrote the paper. ÅO: additional growth rate assays. All authors contributed to the article and approved the submitted version.

## Funding

This work was supported by the Carl Tryggers Foundation for Scientific Research.

## Conflict of Interest

The authors declare that the research was conducted in the absence of any commercial or financial relationships that could be construed as a potential conflict of interest.

## Publisher's Note

All claims expressed in this article are solely those of the authors and do not necessarily represent those of their affiliated organizations, or those of the publisher, the editors and the reviewers. Any product that may be evaluated in this article, or claim that may be made by its manufacturer, is not guaranteed or endorsed by the publisher.
