# Supplementary material for: Mitonuclear Genetic Interactions in the Basidiomycete Heterobasidion parviporum Involve a Non-conserved Mitochondrial Open Reading Frame
Source: Front Fungal Biol. 2021 Dec 14;2:779337. doi: 10.3389/ffunb.2021.779337 (PMC10512249; doi:10.3389/ffunb.2021.779337)
Supplement: Supplementary file 6 [file Data_Sheet_1.docx]

**Supplementary Methods S3: Filtering and analysis of nuclear SNPs**

- All Perl scripts in **bold** in the text below are available on [Github under repository CytN](https://github.com/phcler/CytN).
- All intermediate data files underlined are stored together in Supplementary Results file S4.
- Source FASTA sequence files (*H. parviporum* reference genome sequence, *H. irregulare* transcript sequences) and vcf files resulting from SNP calling in *H. parviporum* have been already published and are available on request (Olson et al. 2012; Clergeot et al. 2019).

1. SNP allelic filtering

First step of the procedure was to select SNPs fulfilling the allelic combination required, using Perl script **PHC_CytN_01.pl**.

In this script, allelic selection is encoded by a string where "0" stands for the reference allele, "1" for the alternative allele and "\d" for a digit (either 0 or 1). The order of characters in this string follows the order of the 30 homokaryons in the vcf files resulting from parallel SNP calling (Clergeot et al. 2019; see Supplementary Material file S1, thumbnail "Labels", column "SNP calling order"). In the context of this work, one allele is borne by homokaryotic isolates Ho9 and Ho32, the other borne by Ho2, Ho5, Ho11, Ho18, Ho19, Ho20, Ho27, Ho26, Ho30 and Ho31, and is undetermined for all other 18 isolates. As Ho18 was the reference isolate for SNP calling (Clergeot et al. 2019), Ho9 and Ho32 are therefore bearing the alternative allele. As a result, the character string chosen for allelic SNPs selection is:

00\d0\d\d\d\d\d\d\d0\d\d10\d0000\d\d\d\d\d\d\d10

This script does not only select SNPs *sensu stricto*, but also biallelic indels.

The following bash command calls all vcf files one by one, process them with script **PHC_CytN_01.pl**, and compiles the filtered SNPs in a single output file named snp_9cytn32_01.txt:

while read line;

do (./PHC_CytN_01.pl <"$line" >> snp_9cytn32_01.txt);

done <numerically_sorted_vcf_files_30_isolates_list.txt

The input file contains a list giving the full path to each vcf file resulting from SNP calling, one path per line. For example, the first line is:

parallel_snp_calling/parallel_snp_call_29_isolates_and_ref_vcf_files/1_parallel_snp_call_29_isolates_and_reference.vcf

Three filtering steps were subsequently implemented using Perl scripts as follows:

SNPs were selected only if:

1) their number of reads corresponding to the reference allele and to the alternative allele were both different from 0 in each isolate, using Perl script **PHC_GenDist_02.pl**;

2) their genotype was supported by more than 90% of the reads in each isolate, using **PHC_GenDist_03.pl**;

3) they were not found in two unitigs bearing the mitochondrial genome sequence, using Perl script **PHC_GenDist_05.pl** (output file of the three steps: snp_9cytn32_02.txt).

The resulting file was converted to a simplified Hapmap format using Perl script **PHC_CytN_02.pl** (output file: snp_9cytn32_03.txt). SNPs were subsequently filtered for a QUAL phred-scale quality score above 100 using Perl script **PHC_CytN_03.pl** (output file: snp_9cytn32_04.txt).

In this list containing 1242 SNPs, each SNP is displayed on a single line with the following information: Q score, reference allele/alternative allele, unitig number, position, allele borne by each isolate, all separated by tabs. For example:

2031.52 G/A unitig_4 12853 G G G G G G G G G G A G G A A G G G G G G G G G G G G G A G

2. Characterization of filtered SNPs loci

An automated procedure was designed to find out whether or not filtered SNPs are: 1) localized into the open reading frame of a gene in *H. parviporum*; 2) causing a non-synonymous mutation. This procedure relies on standalone nucleotide blast searches with *H. parviporum* genomic fragments surrounding each SNP as queries, and a custom database including all *H. irregulare* open reading frames (ORF) as subjects. Blast results files were subsequently analyzed with a set of Perl scripts to detect non-synonymous mutations.

2.1 Creation of a *H. irregulare* ORF database

Blast programs were downloaded and installed according NCBI instructions (https://blast.ncbi.nlm.nih.gov/). A *H. irregulare* ORF database was created from a list of 13405 transcript sequences (Olson et al. 2012). ORF prediction was performed for each transcript sequence using Perl script **PHC_CytN_04.pl**. All ORF sequences were compiled in a single file in FASTA format. The name of each sequence was shortened to the minimum using Perl script **PHC_CytN_05.pl** in order to accommodate the NCBI script for the creation of a database (output file: Hirreg_ORFs.fsa). A nucleotide blast database was then created using the following command:

makeblastdb -in Hirreg_ORFs.fsa -parse_seqids dbtype nucl

2.2 Creation of a list of *H. parviporum* genomic query sequences

Perl script **PHC_CytN_06.pl** was designed to retrieve a piece of genomic DNA sequence surrounding each SNP. The fasta file of *H. parviporum* reference genome sequence was used as input (Clergeot et al. 2019). The SNP position, the name of the unitig bearing it and the length of the piece desired are given on the command line for the script to run. For example, with the SNP cited above, the command to get a 200 bp genomic fragment with this SNP located in its middle is:

./PHC_CytN_06.pl 12853 unitig_4 200

The output is then (with SNP position in bold and underlined):

ACATCTTCAATACTCCATGTCTACTAATGAAACATTCAGGATGTCATATTGAATAGCTTTTGTATTTATTGATTCAGACGGAATTGTGTGGAATTTCAA**G**TTTGGCTCCCAAAACCCAGACTGTGCGAAGCACAGTCTGGTTTTGAGGGGTAGACTGTTTAAATCAATATCTATTGAACCCATGATTCAATCTCACTGTT

A list of the positions and unitig names of the 1242 SNPs filtered above was created using Perl script **PHC_CytN_07.pl** (output file: snp_9cytn32_05.txt). The same was done to create a list of the two alleles of each SNP with Perl script **PHC_CytN_08.pl** (output file: snp_9cytn32_06.txt). Then genomic fragments surrounding each SNP were retrieved and saved in separate FASTA files using the following command:

i=1;

while read line;

do (./PHC_CytN_06.pl $line 800 >

snp_9cytn32_800bp_genomic_fasta_files/snp_9cytn32_800bp_genomic_seq$i.fsa);

let "i+=1";

done <snp_positions_9cytn32.txt

2.3 Nucleotide blast searches

In order to carry out nucleotide blast searches with all SNP loci simultaneously, the basic command - sent from the folder where the blast program is installed - is:

i=1;

while read line;

do (blastn

-db /complete_path_to/Hirreg_ORFs_database/Hirreg_ORFs.fsa -query /complete_path_to/ snp_9cytn32_800bp_genomic_fasta_files/snp_9cytn32_800bp_genomic_seq$i.fsa

-out /complete_path_to/Blast_results/blast_snp_9cytn32_800bp_genomic_vs_ORFs/$i.out);

let "i+=1;

done </complete_path_to/snp_9cytn32_05.txt

Blast results files were thus saved under the number corresponding to the rank of each SNP in the filtered list (see file snp_9cytn32_04.txt above), plus the suffix ".out". If a hit was found, the file displays some text information about the search (including the *H. irregulare* ORF hit, preceded by ">") and one or more local DNA alignments.

Several tests were carried out to optimize genomic fragment length and nucleotide blast options (see below "Analysis of nucleotide blast results"). Increasing the length of the genomic fragment helps, but without significant improvement beyond 800 bp. The best nucleotide blast options found for this analysis are the following:

-word_size 10 -reward 1 -penalty -1 -gapopen 4 -gapextend 1

-min_raw_gapped_score 30

See additional explanations about these options on the NCBI [webpage](https://www.ncbi.nlm.nih.gov/books/NBK279684/).

2.4 Analysis of nucleotide blast results

Preliminary comment

The analysis of nucleotide blast results has to face three challenges:

1) blastn is not a pairwise DNA sequence alignment program. Most of the time, results consist in a fragmented and incomplete alignment between Subject (a *H. irregulare* ORF) and Query sequences (a piece of *H. parviporum* genomic DNA);

2) The Subject sequence is an ORF, whereas the Query sequence is genomic, meaning that introns in the Query sequence will not align;

3) *H. irregulare* and *H. parviporum* are closely related species. However, a few genes in one species may lack in the other, and some other genes may have a relatively low similarity.

All these issues can be properly addressed with an analytic script if one keeps in mind that the most important thing is not to lose track of the reading frame of the Subject sequence (*H. irregulare* ORF). The start codon of the Subject sequence may not be present in the fragments aligned in a blast result file, but this file always keeps track of the position of any fragment of the Subject sequence in relation to the start codon. Even though *H. irregulare* and *H. parviporum* are different species, they are close enough to assume that, for the vast majority of their genes, the reading frame is conserved between orthologues. Therefore, there is enough information in a blast result file to infer whether or not a SNP might cause a non-synonymous mutation in almost any gene in *H. parviporum*.

2.4.1 Identification of SNPs localized into a *H. parviporum* exon

Development of the first analytical Perl script **PHC_CytN_08.pl** was made in parallel with testing for finding the optimal length for the Query sequence and the best parameters for nucleotide blast search (see above), this mainly to address the problem of correct intron detection. However this script leaves deliberately some cases for manual check when they are too risky or to complex to solve automatically, especially if they involve a frame shift.

The script requires two arguments to run: SNP position within the Query sequence (400 for a genomic fragment of 800 bp), and maximum number of codons potentially modified by the substitution of the reference allele by the alternative allele. For example, if the reference allele is GCTG and the alternative allele is ACTA, this number is 2. For the processing of a large number of blast result files together, it is safer not to set this value lower than 4.

The script has several possible outputs. They are all starting with the name of the input blast result file (for example "4.out") and the name of the Subject sequence (for example ">451528") if a hit was found in *H. irregulare* ORF database:

- "**no hit found**" means that the blastn program has not found any *H. irregulare* ORF similar to this *H. parviporum* genomic Query sequence;

- "**alignment score too low**" means that the similarity is too low (cutoff = 180) to build any convincing alignment between Query and Subject sequences;

- "**snp in 3' (or 5') UTR**" means that the SNP was found in the 3' (or 5') untranslated region downstream (or upstream) of the coding sequence of a *H. parviporum* gene which has an orthologue in *H. irregulare*;

- "**snp into intron**" means that the SNP was found in an intron of a *H. parviporum* gene which has an orthologue in *H. irregulare*;

- "**cds frameshift**" means that the SNP was possibly found in the coding sequence of a *H. parviporum* gene which has an orthologue in *H. irregulare*, but that a gap in the alignment of the two sequences results in a frame shift. As the alignment resulting from a nucleotide blast is rarely complete, it requires a manual check with a DNA editor to confirm such a frame shift or not;

- "**cds manual check required**" means that the SNP was possibly found in the coding sequence of a *H. parviporum* gene which has an orthologue in *H. irregulare*. However, it could not be ascertained whether or not it is in an exon or an intron and requires a manual check with a DNA editor to confirm;

- If a SNP is undoubtedly found in an exon of a gene of *H. parviporum* which has an orthologue in *H. irregulare*, the output will be, for example:

cds 738 T plus 2219 2 740 CTACACAATCGC

"**cds**": SNP is into an exon; "738": score of the alignment between Query and Subject sequences; "T": nucleotide at the position expected for the SNP in the Query sequence; "plus": strand on which the Query sequence aligns with the Subject sequence; "2219": position in the Subject sequence corresponding to the SNP in the Query sequence; "2": frame used to translate the Query sequence in order to align with the translation of the Subject sequence; "740": codon in the Subject sequence corresponding to the codon bearing the SNP in the Query sequence; "CTACACAATCGC": codons in the Query sequence potentially modified by SNP allele substitution (here four).

All blast result files were analyzed simultaneously using the following commands:

for file in *.out;

do (printf "$file\t" >> snp_9cytn32_07; ./ PHC_CytN_08.pl 400 4 <$file >> snp_9cytn32_07);

done

and:

sort -n < snp_9cytn32_07.txt > snp_9cytn32_08.txt

For the 1242 SNPs filtered above, the repartition between the different possible outputs is:

no hit found: 277

alignment score too low: 222

cds - frame shift detected: 30

cds - manual check required: 30

3' or 5' UTR: 151

intron: 104

coding sequence: 428

Further investigations (not shown here) have demonstrated that, in many cases, a low alignment score results from the fact that the overlap between Query and Subject sequences is too short. Beyond 800bp, increasing genomic fragment length of the Query has for only consequence to shift some SNPs from the category "no hit found" to "alignment score too low", or from "alignment score too low" to "3' or 5' UTR".

For each SNP requiring a manual check after this step (60 out of 1242: 4,8%), a DNA editor (SerialCloner v2.6.1) was used to align the *H. irregulare* ORF found by nucleotide blast search to its *H. parviporum* orthologue, splice introns *in silico* if needed, rule out a frame shift or not if required, and to localize the SNP position and codon(s) involved if applicable. For very large coding sequences bearing many SNPs, having to reconsider just one manually may have consequences for all of those found in this coding sequence. The file resulting from the analysis of the blast results was then modified accordingly to give:

> snp_9cytn32_09.txt

After manual checks, output repartition of the 1242 SNPs is:

no hit found: 277

alignment score too low: 214

frame shift: 3

no reliable ORF: 15

3' or 5' UTR: 154

intron: 130

coding sequence: 449

2.4.2 Identification of SNPs causing non-synonymous mutations in *H. parviporum*

A second analytical Perl script, **PHC_CytN_09.pl** was designed to this end. It sorts out SNPs localized into an exon and uses the information about the Query sequence to predict which type of mutation the alternative SNP allele might cause. This script requires the rank of each SNP in the list of SNP filtered (file snp_9cytn32_04.txt; see paragraph 1 above) and both its alleles to run. This information was provided by file snp_9cytn32_06.txt (see paragraph 2.2 above) through the following command:

while read line;

do (./PHC_CytN_09.pl $line >> snp_9cytn32_10.txt);

done <snp_9cytn32_06.txt

A typical output line of this command looks like this:

35 >451528 + CTA 2 L CCA P non-synonymous 1 1

"35": rank in the list of filtered SNPs; ">451528": identification of *H. irregulare* ORF found by nucleotide blast search; "+": strand of the *H. parviporum* genomic fragment aligning with this ORF; "CTA": codon bearing the SNP reference allele in *H. parviporum*; "2": position of the SNP in this codon; "L": amino-acid translation of the reference codon; "CCA": same codon bearing the SNP alternative allele; "P": translation of the alternative codon; As these codons do not encode the same amino-acid, the SNP is causing a "non-synonymous" mutation in the exon of all *H. parviporum* isolates bearing the alternative allele; "1 1": both SNP alleles do not affect more than one codon (if the alleles were AT/ATGT, the output would be "2 2").

Perl script **PHC_CytN_10.pl** was run to separate information about SNPs causing non-synonymous mutations from the one about SNPs causing synonymous mutations (output file: snp_9cytn32_11.txt). The resulting file contains 158 SNPs, covering 78 putative ORFs in *H. parviporum*.

2.4.3 Collection of information about the allele borne by each isolate of the SNPs causing non-synonymous mutations

Once SNPs causing non-synonymous mutations were identified, the information stored about them in file snp_9cytn32_04.txt was gathered in two steps as follows:

1) Their positions and unitig names in *H. parviporum* reference genome sequence was recorded using Perl script "**PHC_CytN_11.pl**" and the following command:

i=1;

while read line;

do (./PHC_CytN_11.pl $i $line >>snp_9cytn32_12.txt);

let "i+=1";

done <snp_positions_9cytn32.txt

2) Their full information was then collected using Perl script "**PHC_CytN_12.pl**" and the following command:

while read line;

do (./PHC_CytN_12.pl $line >> snp_9cytn32_13.txt);

done < snp_9cytn32_12.txt

The resulting file was copied and pasted into an Excel Sheet, together with the content of snp_9cytn32_10.txt file. An additional column was added to this sheet ("Alt allele") to count, for each SNP, how many of the 30 homokaryotic isolates are bearing the alternative allele (using Excel function COUNTIF). All lines were subsequently sorted in ascending order according to these counts (see Supplementary Data file S6, thumbnail "Nuclear SNPs (non-syn)").

Additional information about the proteins translated from the 78 genes identified was collected at NCBI. Accession numbers to their mRNA webpages at NCBI were included in a separate column.

2.4.3 SNPs localized into a *H. parviporum* intron or gene regulatory sequence (promoter, 5'UTR, 3'UTR)

Analytical Perl script "**PHC_CytN_13.pl**" was designed to retrieve the information about SNPs localized in a *H. parviporum* intron from file snp_9cytn32_09.txt. Each line of the output (snp_9cytn32_14.txt) displays the identification number of the *H. irregulare* transcript corresponding to a *H. parviporum* gene in which SNPs were found in an intron, and the number of SNPs in that situation.

For example:

384409 4

means that 4 of the 1242 SNPs from snp_9cytn32_09.txt were found in one or more intron(s) of the *H. parviporum* gene orthologous to *H. irregulare* transcript number 384409.

The last line of the output gives the number genes in which one SNP or more were found in an intron: 52 genes for the 130 SNPs previously identified in a intron (see above description of snp_9cytn32_09.txt).

This script was slightly modified to retrieve similar information about SNPs in gene regulatory sequences from the same file. For SNPs located between the start codon of a *H. parviporum* gene and the nucleotide 800 bp upstream, line #9 of the script's code

if ($lines[$i] =~ /intron/) {

was replaced by

if ($lines[$i] =~ /5'UTR/) {

The output was named snp_9cytn32_15.txt.

Similarly, for SNPs located between the stop codon of a *H. parviporum* gene and the nucleotide 800 bp downstream, line #9 of "**PHC_CytN_13.pl**" was replaced by:

if ($lines[$i] =~ /3'UTR/) {

The output was named snp_9cytn32_16.txt.

Analytical Perl script "**PHC_CytN_14.pl**" was designed to create a file containing the identification number and DNA sequence of each *H. irregulare* transcript from either file snp_9cytn32_14.txt (intron), snp_9cytn32_15.txt (5'UTR) or snp_9cytn32_16.txt (3'UTR) using the following command:

while read line;

do (./PHC_CytN_14.pl $line < Hirreg_ORFs.fsa >> snp_9cytn32_17.txt);

done < snp_9cytn32_14.txt

Similarly, output files snp_9cytn32_18.txt and snp_9cytn32_19.txt were created from files snp_9cytn32_15.txt and snp_9cytn32_16.txt respectively.

Additional information about the proteins translated from genes identified this way was collected at NCBI. Accession numbers to their mRNA webpages at NCBI were collected when their annotation pointed at a mitochondrial function. Information about all SNPs localized in a *H. parviporum* intron or gene regulatory sequence were compiled in Supplementary File S6, tab "Nuclear SNPs (All)".
