# Supplementary figures and images for: Mitonuclear Genetic Interactions in the Basidiomycete Heterobasidion parviporum Involve a Non-conserved Mitochondrial Open Reading Frame
Source: Front Fungal Biol. 2021 Dec 14;2:779337. doi: 10.3389/ffunb.2021.779337 (PMC10512249; doi:10.3389/ffunb.2021.779337)

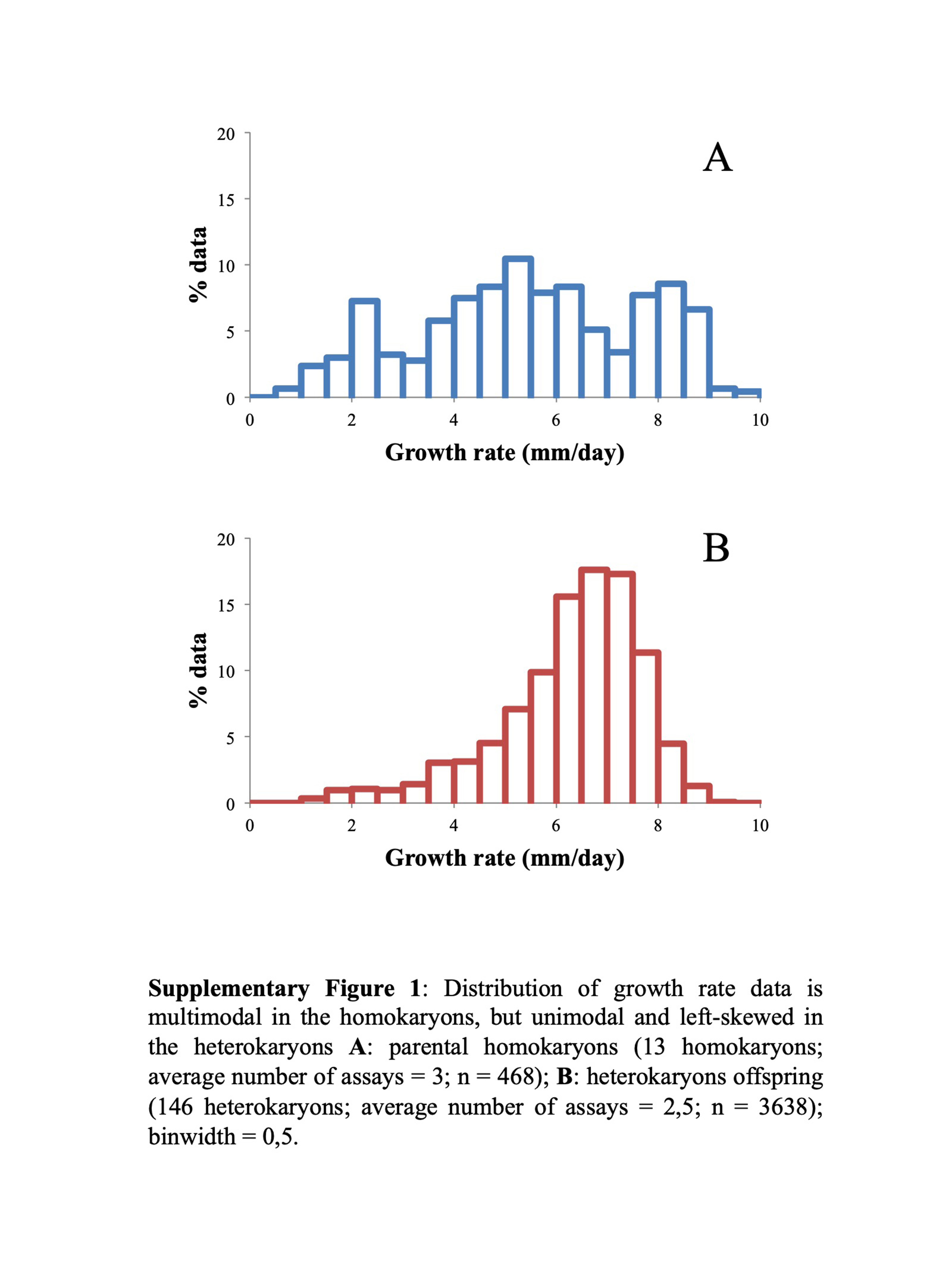

Supplement: Supplementary file 8 [file Image_1.JPEG]
